# Medical and Philosophical News

**Published:** 1781-01

**Authors:** 


At the anniverſary meeting of the Medical Society of London, on the 18th of January, the following gentlemen were elected officers of the Society for the year enſuing; Dr. Simmons, F. R. S. Preſident; Dr. Lettſom, F. R. S. Treaſurer; Dr. Hulme, Librarian; Dr. Kooſtray, Dr. Wood, and Mr. French, Secretaries. On this occaſion an oration in Latin, on the beſt means of proſecuting medical enquiries, was delivered by Dr. Simmons.

